# Editorial: Mechanistic insight and therapeutic potential for the management of non-alcoholic steatohepatitis (NASH)

**DOI:** 10.3389/fendo.2024.1503460

**Published:** 2024-11-11

**Authors:** Umashanker Navik, Amit Khurana, Jasvinder Singh Bhatti

**Affiliations:** ^1^ 1Department of Pharmacology, Central University of Punjab, Bathinda Punjab, India; ^2^ Laboratory of Translational Medicine and Nanotherapeutics, Department of Human Genetics and Molecular Medicine, School of Health Sciences, Central University of Punjab, Bathinda, India

**Keywords:** non-alcoholic steatohepatitis (NASH), genistein, yes-associated Protein (YAP), liver enzyme markers, glucose-to-albumin ratio

The growing incidence of metabolic disorders like non-alcoholic fatty liver disease (NAFLD) and non-alcoholic steatohepatitis (NASH, now renamed MASH) has sparked rising interest in identifying novel prognostic and diagnostic markers that will enhance therapeutic strategies. This Research Topic brings together five key studies contributing to our understanding of NAFLD, or MASLD, and related metabolic conditions. These studies underscore the complex interactions between molecular and metabolic pathways, offering fresh insights into gender-specific treatments, hypoxia-driven NAFLD and fibrosis, and novel predictive tools for early detection. From examining the sex-specific effects of genistein in mitigating obesity and insulin resistance to exploring the potential of new biomarkers and indices, this article emphasizes the importance of targeted, personalized approaches. Together, these studies demonstrate pivotal mechanisms underlying metabolic dysfunction and provide valuable perspectives for improving the diagnosis and management of NAFLD.

Kositanurit et al. investigate the sex-specific effects of plant-derived isoflavone, genistein, on the mitigation of obesity and metabolic dysfunction in gonadectomized mice. The gonadectomized mice serve as a model for individuals experiencing hormonal changes related to metabolic dysfunction, such as post-menopausal women or aging men. Their research highlights the ability of genistein to reduce obesity, hepatic steatosis, and insulin resistance, with significant benefits observed in female mice. This study provides valuable insights into the potential role of genistein as an alternative therapeutic option for individuals having depleted levels of sex hormones and suffering from obesity, insulin resistance, and metabolic dysfunction-associated steatotic liver disease (MASLD) (1). Yan et al. explore the role of hypoxia-inducible factor-2α (HIF-2α) in exacerbating fibrosis in non-alcoholic fatty liver disease (NAFLD). This study demonstrates the role of chronic hypoxia, which upregulates HIF-2α activation, leading to the promotion of yes-associated protein (YAP) and inhibition of YAP-phosphorylation, which in turn enhances the NAFLD progression through glutaminolysis-induced hepatic stellate cells activation and collagen deposition. This study presents new opportunities for treating NAFLD and its progression in populations exposed to chronic hypoxia, such as those living at high altitudes. Predictive tools that can identify non-obese people with NAFLD remain under investigation (Zheng et al.). However, rigorous studies will prospectively evaluate any novel index against validated measures longitudinally. Further studies are required, with careful anthropometric estimates that consider body fat distribution through accepted and validated anthropometric estimates of central obesity, such as waist circumference. Qiu et al. present a gender-focused analysis comparing the associations between liver enzyme markers alanine aminotransferase (ALT), aspartate aminotransferase (AST), and gamma-glutamyl transferase (GGT) and NAFLD. Their study reveals significant gender-specific differences in the correlation between these enzymes and NAFLD, with ALT emerging as a particularly effective marker for screening the disease, especially in males. This finding underscores the accuracy of NAFLD screening, as it suggests that gender-specific approaches may enhance diagnostic effectiveness. The research highlights the need to consider gender-specific approaches during screening of NAFLD. Lastly, Wang et al. investigate the serum levels of glucose and albumin as their ratio (glucose-to-albumin ratio, GAR) relationship with NAFLD and its progression in non-diabetic individuals. Their work demonstrates a positive correlation between higher GAR and increased NAFLD risk, along with its progression into liver fibrosis. This research highlights GAR as a promising prognostic marker for identifying NAFLD and predicting disease progression, providing an important tool for early diagnosis and management of NAFLD.

Together, these articles emphasize the growing body of knowledge around metabolic disorders, and the critical markers that can aid in early diagnosis, treatment, and management. With new insights into sex-specific treatments, hypoxia-induced liver damage mechanisms, and novel predictive indices, these research advances our understanding of NAFLD and related metabolic dysfunctions, offering fresh perspectives for the early diagnosis and therapeutic strategies, which could significantly improve outcomes for individuals at the risk of onset of NAFLD.

