# Corrigendum: Editorial: Mechanistic insight and therapeutic potential for the management of non-alcoholic steatohepatitis (NASH)

**DOI:** 10.3389/fendo.2024.1528628

**Published:** 2024-12-03

**Authors:** Umashanker Navik, Amit Khurana, Jasvinder Singh Bhatti

**Affiliations:** ^1^ Department of Pharmacology, Central University of Punjab, Bathinda Punjab, India; ^2^ Laboratory of Translational Medicine and Nanotherapeutics, Department of Human Genetics and Molecular Medicine, School of Health Sciences, Central University of Punjab, Bathinda, India

**Keywords:** non-alcoholic steatohepatitis (NASH), genistein, yes-associated Protein (YAP), liver enzyme markers, glucose-to-albumin ratio

In the published article, there was an error in the affiliation and correspondence author. Instead of

**“**Umashanker Navik*, Amit Khurana and Jasvinder Singh Bhatti

Department of Pharmacology, Central University Punjab, Bathinda Punjab, India

*CORRESPONDENCE

Umashanker Navik, usnavik@gmail.com”, it should be

“Umashanker Navik^1*^, Amit Khurana^1^ and Jasvinder Singh Bhatti^2*^

^1^Department of Pharmacology, Central University of Punjab, Bathinda Punjab, India

^2^Laboratory of Translational Medicine and Nanotherapeutics, Department of Human Genetics and Molecular Medicine, School of Health Sciences, Central University of Punjab, Bathinda, India.

*CORRESPONDENCE

Umashanker Navik, usnavik@gmail.com

Jasvinder Singh Bhatti, jasvinder.bhatti@cup.edu.in”.

The authors apologize for this error and state that this does not change the scientific conclusions of the article in any way. The original article has been updated.

